# Multimodal genomic prediction is not a buzzword: why modern plant breeding must integrate genomics, enviromics, and phenomics

**DOI:** 10.1093/g3journal/jkag110

**Published:** 2026-06-01

**Authors:** José Crossa, Abelardo Montesinos-López, Jaime Cuevas, Osval A Montesinos-López, Paulino Pérez-Rodríguez, Alison Bentley

**Affiliations:** Programa de Estadística y Ciencia de Datos, Colegio de Postgraduados, Montecillo Texcoco, Estado de México 56230, México; Centro Universitario de Ciencias Exactas e Ingenierías (CUCEI), Universidad de Guadalajara, Guadalajara, Jalisco 44430, México; División de Ciencias, Ingeniería y Tecnologías (DCIT), Universidad Autónoma del Estado de Quintana Roo, Chetumal, Quintana Roo 77019, México; Facultad de Telemática, Universidad de Colima, Colima, Colima 28040, México; Programa de Estadística y Ciencia de Datos, Colegio de Postgraduados, Montecillo Texcoco, Estado de México 56230, México; CSIRO Agriculture and Food, Black Mountain, Canberra, ACT 2601, Australia; Research School of Biology, Australian National University, Canberra, ACT 2601, Australia

**Keywords:** genomic prediction, multimodal, environments

## Abstract

Plant breeding has entered a new era, transitioning from a predominantly unimodal, genomics-focused paradigm to one defined by multimodal data integration. Dense genomic markers, long-term environmental datasets, physiological and omics measurements, and high-throughput phenotyping platforms are now routine components of modern breeding programs. Paradoxically, while plant breeding now operates in a multimodal data ecosystem, most prediction models remain effectively unimodal, relying on a single type of information at a time—genomics alone, enviromics alone, physiology alone, or phenomics alone. This overlooks a fundamental biological reality: complex traits emerge from the interaction of genetic potential, environmental conditions, and dynamic physiological responses. Across the life sciences, the integration of heterogeneous biological data has become a central strategy for improving prediction and discovery. We argue that genomic prediction must become truly multimodal to reflect the biological reality that complex traits emerge from interacting genetic, environmental, and physiological processes. We clarify multimodality as distinct from commonly conflated concepts such as multi-environment or multi-trait prediction and outline strategies for integrating heterogeneous data sources using multi-kernel and multi-stream modeling frameworks.

## Teaser

Genomic prediction models remain essentially unimodal, relying on a single source of information at a time—genomics alone, enviromics alone, or phenomics alone. This practice overlooks a fundamental biological reality: complex traits emerge from the interaction of genetic potential, environmental forcing, and dynamic physiological responses. Here, Crossa *et al*. argue that multimodal genomic prediction is not a fashionable extension of machine learning, but a conceptual necessity for predictive breeding under climate variability.

## A data-rich era limited by unimodal prediction

Modern plant breeding operates in an unprecedented data-rich context. Genome-wide SNP markers are dense, affordable, and routinely generated across crops and breeding programs worldwide (Meuwissen et al. 2001). At the same time, environmental datasets and crop modeling frameworks allow the reconstruction of localized daily weather profiles aligned with crop phenology, enabling the derivation of biologically meaningful environmental covariates that describe stress accumulation, temperature dynamics, radiation balance, and water availability. In parallel, high-throughput phenotyping platforms have transformed field experimentation, providing repeated measurements of canopy temperature, reflectance, plant architecture, and spectral signatures across thousands of plots, environments, and time points (Araus and Cairns 2014). Moreover, the increasing availability of molecular phenotypes from -omics technologies—transcriptomics, proteomics, and metabolomics—has greatly expanded the depth of information, giving rise to a need for innovative and creative methodologies to integrate these data into a comprehensive biological modeling framework.

Together, these developments have led to a truly multimodal data ecosystem in plant breeding. Genomics captures the inherited genetic potential of individuals; enviromics characterizes environmental drivers and stresses; omics captures the molecular phenotypes; and phenomics reflects the dynamic physiological state of the plant as it responds to its surroundings. Transcriptomics provides insight into the gene expression patterns and gene × gene interactions that underpin molecular responses, while proteomics reveals the functional proteins that execute biological processes, and metabolomics captures biochemical activities. Together, these technologies provide a window into the link between phenotype and environmental adaptation. The integration of complementary data layers enables a comprehensive understanding of the genotype–environment–phenotype continuum and results in biologically informed prediction models.

Despite the availability of multimodal data, prediction models used in practice lag behind the available data remaining largely unimodal in both structure and interpretation. Integrative strategies are emerging in agricultural research and are common in commercial breeding programs where genomic, environmental, phenotypic, and management data are combined to accelerate genetic gain and improve decision-making. These developments reflect a broader scientific transition: predictive model accuracy depends on representing biological systems as interacting layers of information rather than isolated data sources (eg Jumper et. al. 2021).

The current genomic prediction models frequently undertake parallel analyses, descriptive summaries, or post hoc interpretations of enviromic and phenomic information rather than integrating the myriad data modalities into unified predictive frameworks.

In many multi-environment trials, environmental variance makes at least an equal contribution to trait variability as genetic variance, particularly under climate stress and across highly heterogeneous target populations of environments (Crossa et al. 2022). When prediction models focus only on genomic effects, environmental effects can be incorrectly reflected in genomic effects, leading to inflated heritability estimates, unstable rankings, and weak prediction especially in novel environments. Conversely, enviromic-only models may describe site quality and variability in exquisite detail but fail to accurately rank genotypes, while phenomic-only models capture transient physiological response without separating inherited potential from short-term stress responses. Each unimodal approach captures part of the system but fails to capture the system itself. The transformative potential of multimodal modeling is illustrated by breakthroughs such as AlphaFold, which combines sequence information, evolutionary signals, and structural constraints within deep learning architectures to accurately predict protein structures (Jumper et al. 2021).

Integrative predictive frameworks are also increasingly implemented in commercial breeding programs, where genomic prediction is combined with crop growth models and environmental covariates to improve selection decisions across target populations of environments (Cooper et al. 2014).

## Multimodality as a biological necessity

Phenotypes are emergent properties of interactions between genetic, environmental, and physiological processes operating across multiple biological layers. Predictive frameworks that fail to represent these interactions are incomplete and likely to underperform. This problem becomes particularly acute under climate change, where nonlinear responses, threshold effects, and phenotypic plasticity and their interactions drive phenotype.

Multi-environment prediction is often labeled as multimodal, yet the modality remains genomic. Multi-trait prediction is similarly mischaracterized, as the underlying data modality remains phenotypic. Simply including genomic markers and environmental covariates into a single design matrix does not constitute multimodality because it does not capture the underlying biological structure of the data; true multimodality requires each data type to be first represented in a form consistent with its biological meaning, and only then be integrated. Genomics, transcriptomics, proteomics, metabolomics, enviromics, phenomics, and pedigree data each have distinct structures; forcing them into a single homogeneous representation of the data structure across modalities risks losing the information that makes each modality valuable.

## Encoding, integration, and evaluation in breeding contexts

A defining feature of genuinely multimodal models is the principle of encoding before integration. Each modality is first transformed into a representation that reflects its biology. Genomic markers generate one modality. Environmental covariates generate a separate modality based on temperature, rainfall, vapor pressure deficit, radiation, or stress indices aligned with crop development. Phenomics yields latent features derived from principal components, partial least squares, or deep learning encoders applied to hyperspectral or thermal time series. Additive relationship matrices derived from pedigree encode known ancestry and historical relatedness. Only after this encoding step are modalities integrated.

The conceptual roots of the multimodal prediction approach in plant breeding can be traced to reaction-norm and genotype × environment interaction models that explicitly combined genomic and environmental information using separate covariance structures (Heslot et al. 2013; Jarquín et al. 2014). These models formalized the idea that genotypic performance depends jointly on genetic relationships and environmental similarity. These joint relationships are modeled as either separate random effects, each with a variance-covariance proportional to a kernel matrix calculated from each mode; or with an integrated random effect where each mode contributes a variance covariance matrix derived from a kernel function. Mixed model data integration techniques have suffered from inappropriate model specification in the hierarchy by including BLUP predictors from one layer into models of other layers (for a description of this issue, see Holland and Piepho (2024)).

While these integrated approaches remain foundational, modern breeding data demand richer and more flexible representations such as those developed within the family of machine learning models commonly referred to as multimodal deep learning. These are specifically designed to integrate heterogeneous data sources while at the same time preserving modality-specific information. Montesinos-López et al. (2024) described that multimodal deep learning, such as deployed by Alpha-fold (Jumper et al. 2021) can integrate heterogeneous data modalities at early, intermediate, or late stages of the modeling pipeline. The choice of integration strategy critically depends on the statistical properties, dimensionality, and reproducibility requirements, and semantic structure of each data source, as well as on the biological or practical meaning of its interactions (Jumper et al. 2021; Montesinos-López et al. 2024; Howard and Lipka 2025).

Integration itself can be performed with different algorithms. Using kernels, for example, multi-kernel averaging, blends complementary similarity structures into a unified predictor. Kernel interaction terms capture genotype-specific sensitivity to environmental stress.

On the other hand, multimodal deep learning architecture preserves separate data-processing pipelines whose layers are fused to learn a shared latent representation that serves as the basis for downstream prediction. In multimodal deep learning models, attention-based mechanisms enable models to adapt the weights of modalities to environmental conditions or phenological stages.

These strategies preserve biological logic while at the same time enabling nonlinear interactions that are essential for modeling complex traits.

Equally important is the way in which predictive performance is evaluated. Breeders must predict new genotypes in known environments, incomplete genotype × environment tables, known genotypes in new environments, and entirely new genotype–environment combinations. While unimodal models often perform adequately within observed environments, they fail in new scenarios because they lack explicit representation of the missing dimension. In contrast, multimodal models are expected to hold the promise of providing more reliable predictions by integrating genetic effects, environmental structure, and additional omics information within a unified framework. Empirical evidence shows superior performance of multimodal frameworks in plant breeding scenarios (Cooper et. al. 2014; Montesinos-López et al. 2019; Costa-Neto et al. 2022).

## Multimodality, climate change, and the future of predictive breeding

Climate change adds urgency to the conceptual shift from unimodal to multimodal modeling. As heat events intensify, rainfall becomes more erratic and environmental conditions depart from historical norms. Phenotypic variance will increasingly reflect physiological plasticity and stress response rather than stable additive genetic effects. Phenomic signals, when integrated with genomic information, provide a window into genotype-specific mechanisms of resilience. Multimodal models are therefore essential for predicting performance under new conditions, designing resilient ideotypes, and sustaining long-term genetic gain for yield and other complex traits.

To appreciate the urgency of this transition, it is useful to reflect on how prediction has historically been framed in plant breeding. Quantitative genetics emphasizes variance partitioning for inference rather than prediction. Early genomic selection models represented a major conceptual advance by shifting the focus toward predicting breeding values using dense marker data. However, these models were developed in a context where environmental information was sparse and phenotyping was limited. Under those conditions, a unimodal genomic framework was both pragmatic and effective. That context has now changed fundamentally.

To achieve the optimal integration of available information into the current landscape of large-scale biological data, novel methodological approaches are required. Multimodal models, particularly deep learning frameworks, offer substantial potential to enable scalable analyses of massive breeding datasets by efficiently processing heterogeneous data sources. This scalability allows the simultaneous evaluation of thousands of candidate lines, thereby optimizing resource allocation and reducing operational costs.

Despite the growing availability of diverse data modalities, their effective integration into breeding programs remains a central challenge. Deep learning models address this limitation by automatically extracting complex and nonlinear representations from raw multi-source data, thereby reducing reliance on manual feature engineering and improving predictive accuracy. Moreover, the optimal integration of big biological data within multimodal deep learning architectures can substantially enhance predictive performance through the integration of complementary information across data sources.

Multimodality should therefore be viewed as a natural evolution of genomic selection rather than a departure from its principles. Importantly, multimodal prediction implies more sophisticated modeling approaches that require expertise in tuning complex models, more computational resources and training data. However, properly constructed multimodal models often yield more stable and interpretable predictions than unimodal alternatives by explicitly separating genetic, proteomics, transcriptomics, metabolomics, environmental, and physiological sources of variation. This separation is not only biologically meaningful but also operationally necessary to enable more efficient modeling and to provide breeders with clearer, more actionable information for making selection decisions.

Multimodal frameworks are also inherently scalable. Not all breeding programs will have access to dense phenomics or exhaustive environmental reconstructions, but multimodality does not require all data layers simultaneously. Models can integrate modalities where available and expand as capacity grows. Even modest environmental covariates, when biologically aligned, can substantially improve predictive robustness. Thus, multimodality should be seen as a flexible framework rather than all-or-nothing requirement. Also, multimodal models can take advantage of transfer learning approaches that may boost prediction accuracy in plant breeding by leveraging knowledge across traits or environments, enabling more efficient and scalable implementation of deep learning in genomic prediction. To fully harness the potential of transfer learning, however, it is essential to develop pretrained models tailored to specific species, traits, and environments. Such specialization will unlock its power to enhance model adaptability, predictive accuracy, and cross-context generalization in genomic prediction.

The transition from unimodal to multimodal prediction is both technical and cultural. This type of change will require breeders, quantitative geneticists, statisticians, physiologists, and data scientists to move beyond familiar but increasingly inadequate paradigms. It also requires journals, reviewers, and funding agencies to recognize that biologically motivated model complexity can be a strength rather than a liability.

From accumulated experience, several principles for robust multimodal pipelines emerge. Each modality should be encoded independently before integration. Environmental covariates should be biologically aligned with the crop development time course rather than aggregated over entire seasons. Kernels and latent features must be normalized to avoid dominance by scale. Nonlinear encoders should be favored for modalities exhibiting threshold responses. Fusion strategies should be guided by biological interactions rather than computational convenience. Evaluation schemes must reflect real breeding decisions. Finally, interpretability should be prioritized, whether through kernel weights, modality contributions, variance partitions, attention mechanisms or prediction goals, to maintain trust and insight for breeders.

In conclusion, multimodal genomic prediction should not be viewed as a straightforward extension of existing models or a fashionable trend, but as the foundational paradigm for predictive breeding in the twenty-first century. As environmental variability increases and trait architecture becomes more complex, unimodal frameworks will increasingly fail to meet breeding objectives. Integrating genomics, proteomics, transcriptomics, metabolomics, enviromics, phenomics, pedigree information, and additional omics layers into coherent predictive frameworks is essential for designing robust ideotypes, maintaining genetic gain, and developing climate-smart breeding strategies.

The question is no longer whether multimodal prediction will become necessary in plant breeding, but how quickly the breeding community will adapt to a reality where biological systems must be effectively modeled as interacting layers of information.
